# Supplementary figures and images for: Development of a Fluorescent Tool for Studying Legionella bozemanae Intracellular Infection
Source: Microorganisms. 2021 Feb 13;9(2):379. doi: 10.3390/microorganisms9020379 (PMC7917989; doi:10.3390/microorganisms9020379)

FIG S1.

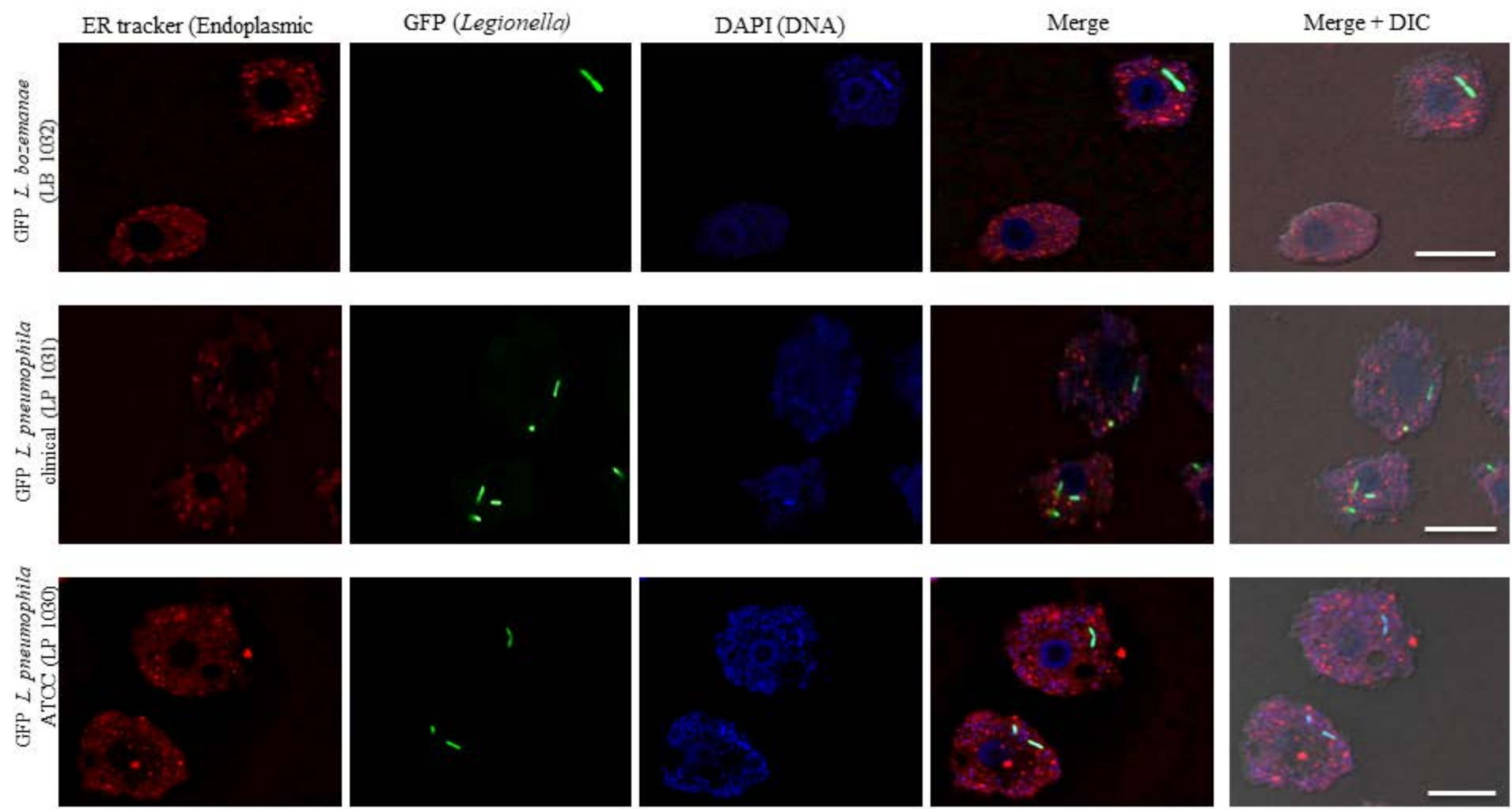

Supplement: Supplementary file 1 [file microorganisms-09-00379-s001.pdf]
